# Histone acetylation dynamics in repair of DNA double-strand breaks

**DOI:** 10.3389/fgene.2022.926577

**Published:** 2022-09-09

**Authors:** Shalini Aricthota, Paresh Priyadarshan Rana, Devyani Haldar

**Affiliations:** Laboratory of Chromatin Biology and Epigenetics, Centre for DNA Fingerprinting and Diagnostics, Hyderabad, Telangana, India

**Keywords:** chromatin, histone acetyltransferase (HAT), histone deacetylase (HDAC), histone modifications, chromatin remodelling, homologous recombination, DNA double-strand break repair pathway choice

## Abstract

Packaging of eukaryotic genome into chromatin is a major obstacle to cells encountering DNA damage caused by external or internal agents. For maintaining genomic integrity, the double-strand breaks (DSB) must be efficiently repaired, as these are the most deleterious type of DNA damage. The DNA breaks have to be detected in chromatin context, the DNA damage response (DDR) pathways have to be activated to repair breaks either by non‐ homologous end joining and homologous recombination repair. It is becoming clearer now that chromatin is not a mere hindrance to DDR, it plays active role in sensing, detection and repair of DNA damage. The repair of DSB is governed by the reorganization of the pre-existing chromatin, leading to recruitment of specific machineries, chromatin remodelling complexes, histone modifiers to bring about dynamic alterations in histone composition, nucleosome positioning, histone modifications. In response to DNA break, modulation of chromatin occurs *via* various mechanisms including post-translational modification of histones. DNA breaks induce many types of histone modifications, such as phosphorylation, acetylation, methylation and ubiquitylation on specific histone residues which are signal and context dependent. DNA break induced histone modifications have been reported to function in sensing the breaks, activating processing of breaks by specific pathways, and repairing damaged DNA to ensure integrity of the genome. Favourable environment for DSB repair is created by generating open and relaxed chromatin structure. Histone acetylation mediate de-condensation of chromatin and recruitment of DSB repair proteins to their site of action at the DSB to facilitate repair. In this review, we will discuss the current understanding on the critical role of histone acetylation in inducing changes both in chromatin organization and promoting recruitment of DSB repair proteins to sites of DNA damage. It consists of an overview of function and regulation of the deacetylase enzymes which remove these marks and the function of histone acetylation and regulators of acetylation in genome surveillance.

## Introduction

Genomic integrity is constantly compromised by DNA damage arising from exposure to endogenous and exogenous genotoxic agents. Double-strand breaks (DSBs) are the most dangerous form of DNA damage that are caused from exposure to ionizing radiation (IR), the collapse of DNA replication forks or during processing of certain types of DNA lesion. If not detected and repaired rapidly, these can cause mutations, chromosomal rearrangements, genomic instability, cell death or diseases like cancer (Jackson and Bartek, 2009; Ciccia and Elledge, 2010; Kieffer and Lowndes, 2022). Two major evolutionarily conserved pathways have evolved to protect organisms from DSB, non-homologous end joining (NHEJ) and homologous recombination (HR). The NHEJ pathway repairs the damaged DNA ends by direct religation, whereas in HR, the intact sister chromatid (present at S-phase and G2 phase) is used as a template for repair (Lieber, 2010; Chapman et al., 2012). However, a fundamental question remains on how one of these specific pathways is chosen although several factors influencing the DNA repair pathway choice such as chromatin structure, DNA end resection, cell cycle phase and transcription have been identified (Chapman et al., 2012; Aymard et al., 2014; Hustedt and Durocher, 2016). Studies over last three decades have shown how cells detect and repair DSBs and established that in addition to the proteins directly involved in DNA repair, chromatin structure surrounding the DSB and the factors regulating it, plays a conserved active role in facilitating DNA damage signalling and repair (Lukas et al., 2011; Soria et al., 2012; Mohan et al., 2021). The ability of cells to mount an effective DNA damage response is regulated by the chromatin dynamics of the region surrounding the DSB.

**FIGURE 1:**
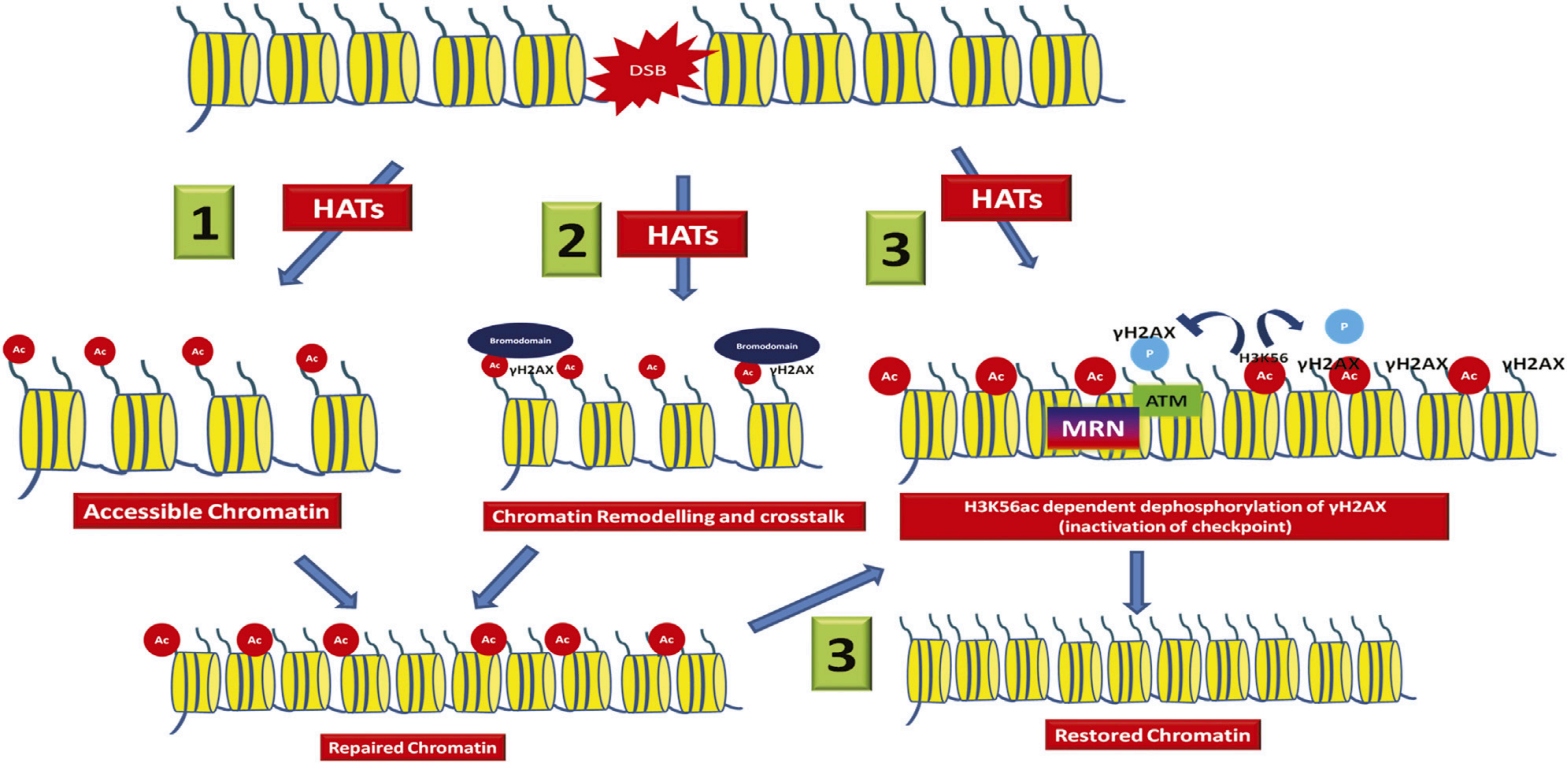
Different roles of histone acetylation at the DSB. At the DSB, acetylation of histones by the action of histone acetyltransferases leads to- 1) Charge based increase in chromatin accessibility leading to recruitment of repair factors. 2) The acetylated histones are recognized by acetyl readers like bromodomain containing proteins, which in turn leads to chromatin remodelling around the break and DDR factor recruitment. 3) Some modifications like H3K56ac helps in inactivation of checkpoint and therefore leads to chromatin restoration to its native state.

In eukaryotic cells, DNA is wrapped into chromatin in the nuclei. Nucleosome, the basic unit of chromatin, is comprised of 147 base pairs of DNA and a histone octamer with two H2A–H2B dimer and one H3–H4 tetramers (Jenuwein and Allis, 2001; Luger et al., 1997). The N- and C-terminal tails of these histone proteins can be post-translationally modified via acetylation, phosphorylation, methylation, SUMOylation, and ubiquitination (Strahl and Allis, 2000; Kouzarides, 2007). The repair of DSB is governed by the reorganization of the pre-existing chromatin, resulting in recruitment of damage sensors and chromatin remodelers to bring about dynamic alterations in histone modifications leading to recruitment of repair proteins (Soria et al., 2012; Wilson and Durocher, 2017; Mohan et al., 2021). In response to DNA break, modulation of chromatin occurs via various mechanisms including post-translational modification of histones. Upon DBS formation, post-translational modifications like phosphorylation, acetylation, methylation and ubiquitylation are known to be induced on specific histone residues near the DSB, which are signal and context dependent (Kouzarides, 2007; Miller and Jackson, 2012; Van and Santos, 2018). DNA break induced histone modifications have been reported to function in sensing the lesion, activating pathways for processing and repair of breaks to maintain genomic integrity. Formation of DSB induces chromatin decondensation, which is evident from the reports showing increased sensitivity of damaged DNA to micrococcal nuclease (Telford and Stewart, 1989). Several studies have shown that dynamic regulation of histone acetylation via histone acetylases and Histone deacetylases play crucial role in regulating chromatin structure flanking the DSB and is required for activation of the DNA damage response and DSB repair. In response to DSBs, formation of open, relaxed chromatin domains occur which are spatially localized to the area surrounding the break (Figure 1). These relaxed chromatin structures are created through the joint action of the chromatin remodellers and histone acetyltransferases (Qi et al., 2016). The resulting destabilization of nucleosomes at the DSB by chromatin remodeller and histone modifiers, is needed for the subsequent recruitment of the DNA repair proteins. The DSBs are then repaired either by non‐homologous end joining and homologous recombination. Histone acetylation increases chromatin assessibility and therefore has been shown to play a positive role in DSB repair pathway. However, there are reports on requirement of HDAC complexes, for efficient DNA repair by NHEJ (Jazayeri et al., 2004; Miller et al., 2010; Miller and Jackson, 2012). Therefore, understanding about chromatin dynamics at DSBs and the precise role chromatin environment plays to influence the process of DSB repair is not fully understood. Further, there is emerging evidence that the different chromatin structures in the cell, such as heterochromatin and euchromatin, utilize distinct remodeling complexes and pathways to facilitate DSB repair (Caridi et al., 2017). Interestingly, the metabolic state of the cell at the time when DSB occur also influence DNA damage signalling and repair (Sivanand et al., 2017; Vadla et al., 2020). The processing and repair of DSB is therefore critically influenced by the nuclear architecture in which the lesion arises. At the damaged DNA, histone acetylation level changes through signal dependent recruitment and regulation of histone acetyltransferases and histone deacetylases which function in coordination with the ATP dependent remodellers. In this review, we will discuss how chromatin architecture of the region where the DSB is localized alters via dynamic changes in histone acetylation to generate a repair conducive platform to maintain genomic integrity.

### DNA damage response

Double-strand breaks can form directly by breakage of both the strands, or collapse of stalled replication forks. DSBs are quickly detected by mobilizing and recruiting the sensing factors to recognize these lesions and activating the DNA damage checkpoint (Smolka et al., 2007). The signaling pathways begins with the activation of sensors ATM, ATR and DNA-PKCs (Matsuoka et al., 2007). The primary mark for DSB is phosphorylation of H2AX, spreads to megabases around the DSB and triggers downstream processes (Rogakou et al., 1998; Rogakou et al., 1999). One of the earliest cellular responses to DSBs is the rapid recruitment of the ATM kinase and phosphorylation of histone H2AX (known as γH2AX) on either side of the DSB which acts a platform for landing of multiple repair factors to the chromatin (Bonner et al., 2008). For example, initial phosphorylation of H2AX (γH2AX) recruits scaffold protein MDC1 (Stucki et al., 2005) forming a docking platform that promote the recruitment and retention of other DNA repair proteins onto the chromatin at DSBs, including the MRN complex, the RNF8 ubiquitin ligase and the BRCA1, Ku70/80, and 53BP1 proteins (Kolas et al., 2007; Mailand et al., 2007; Melander et al., 2008). In response to DSBs, formation of open, relaxed chromatin domains occur which are spatially localized to the area surrounding the break the relaxed chromatin is created through the joint action created through the joint action of the chromatin remodellers and histone acetyltransferases such as SWI/SNF complexes, Tip60, p300, etc., respectively (Papamichos-Chronakis and Peterson, 2013; Qi et al., 2016). The resulting destabilization of nucleosomes at the DSB by chromatin remodeller and modifiers is needed for ubiquitination of the chromatin by the RNF8 ubiquitin ligase, and for the subsequent recruitment of the NHEJ or HR factors.

**FIGURE 2:**
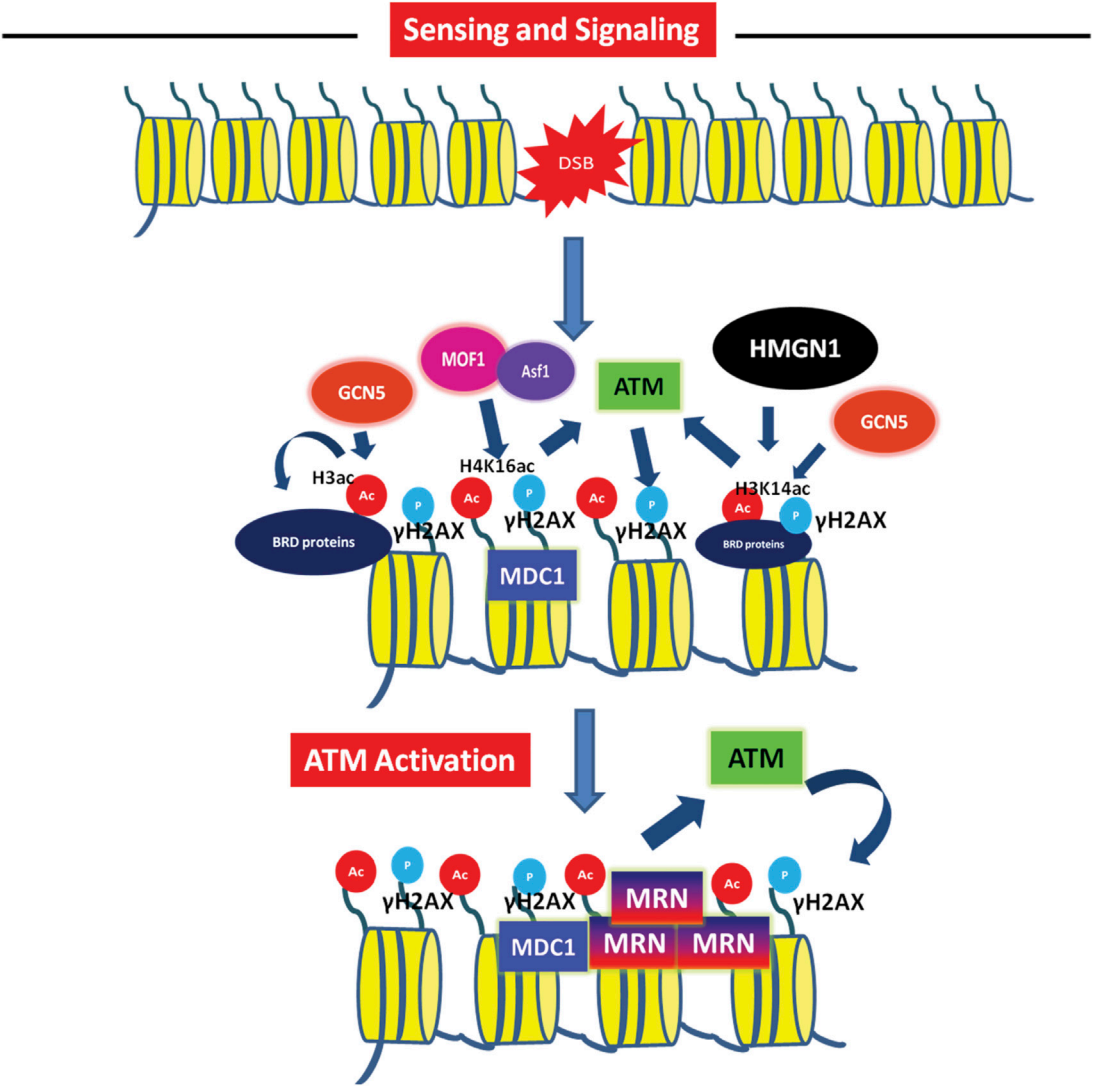
Role of acetylation in sensing and signalling of double-strand breaks Acetylation mediated activation of DNA damage checkpoint leading to DNA damage recognition- The checkpoint sensor ATM phosphorylates H2AX, leading to recruitment of MDC1. This MDC1 recruitment *via* interaction with γH2AX is facilitated by H4K16 acetylation mark established by MOF1 (with help of Asf1 histone chaperon) at the DSB. The H3K14ac by GCN5 and docking of MDC1 promotes ATM activation and spreading of γH2AX mark. This leads to recruitment of pathway specific factors like the MRN complex.

**FIGURE 3:**
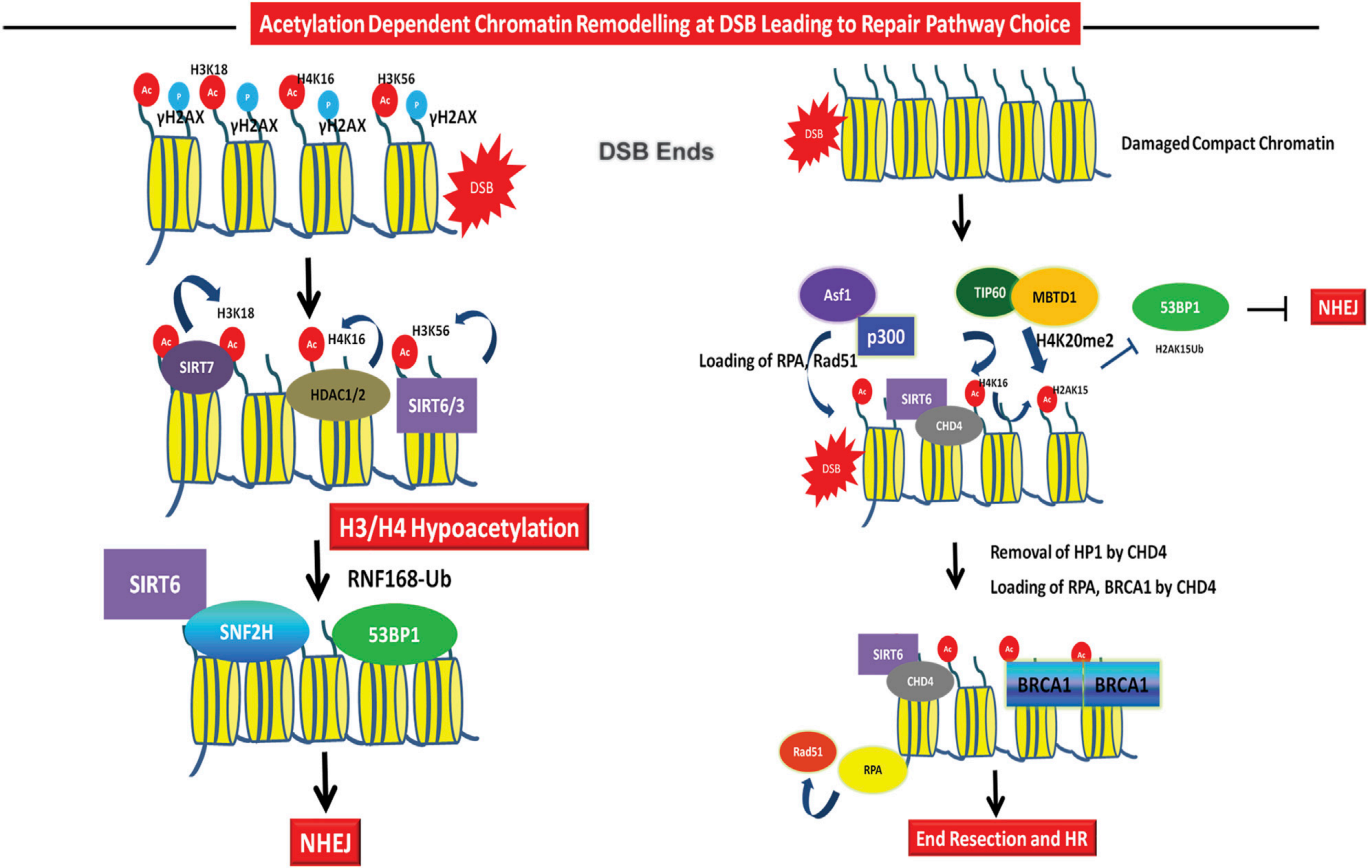
Role of acetylation in DSB repair pathway choice. Repair Pathway Choice- The early recruitment of HDACs like SIRT6, SIRT3, and HDAC1/2 leads to deacetylation of H3K56ac, H3K18ac, H4K16ac, etc. leading to chromatin compaction and recruitment of NHEJ factors 53BP1 and Ku70/80. SIRT6 dependent SNF2H recruitment aids in the recruitment of downstream DNA repair factors at G1 to facilitate NHEJ. The repair pathway choice for HR through acetylation is mediated *via* Tip60 dependent ubiquitylation to acetylation switch at H2AK15, through H4K20me3 leading to inhibitory binding to 53BP1 and inhibition of NHEJ. Repair of damage in G2 or at compact chromatin regions require removal of heterochromatin protein like HP1 by CHD4. CHD4 is recruited by SIRT6 and this leads to removal of HP1 leading to chromatin decompaction, recruitment of RPA and BRCA1 to facilitate HR. Asf1 and p300 also facilitates the recruitment of Rad51 and RPA at DSBs.

The metabolic state and cell cycle stage also affects DSB formation and the response to DSB varies accordingly. Repair via homologous recombination pathway depends on presence of a sister chromatid as template. Hence, the HR pathways is functional during the S/G2 phase, whereas, the NHEJ pathway is active throughout the cell cycle. Recognition of damaged DNA ends by Ku70/80 leads to recruitment of other factors for NHEJ (Kieffer and Lowndes, 2022). Similarly, the HR pathway requires the processing of the DNA by MRN complex and other proteins like RPA, CtIP, Exo1 followed by recruitment of BRCA1 and other HR factors. Checkpoint mediators like 53BP1 of NHEJ pathway and BRCA1 of HR pathways compete against each other to make the pathway choice (Powell and Kachnic, 2003; Panier and Boulton, 2014) (Figure 3 **)**. The repair pathway choice refers to the preference of HR vs. NHEJ pathway for repairing a DSB according to the availability of template DNA and the complexity of the damage (Chapman et al., 2012; Mohan et al., 2021). The γH2AX and the MRN complex is involved in crosstalk with histone modifications for efficient loading of chromatin remodelers and repair factors at the sites of DSBs. The external environment can affect HR machinery via affecting the chromatin modification marks. For example, a low pH environment requires the acetylation level to drop to certain extent for HR to successfully commence upon DSB formation (Vadla et al., 2020). Even the chromatin landscape around a break, like heterochromatin or euchromatin, can influence the repair machinery, calling in the specific repair factors (Caridi et al., 2017; Aleksandrov et al., 2020). The activation of ATM and DNA-PKCs can be influenced by the chromatin remodelers recruited to specific histone marks. There are multiple modes of ATM activation as depicted in Figure 2 and also described in individual histone modifications sections. In addition to these canonical sensors, currently, the role of histone deacetylase SIRT6 has come into light regarding its interaction with CHD4 as a DSB sensor. It involves chromatin relaxation and HP1 release from H3K9me3 for HR machineries to access the damaged DNA (Hou et al., 2020; Meng et al., 2020; Onn et al., 2020), linking heterochromatin regulation to DSB sensing and repair. After the establishment of chromatin marks and recruitment of repair factors, the chromatin remodelers like SWI/SNF and RSC (Remodelling the Structure of Chromatin) complex slide the nucleosomes to make the DNA damage accessible. This demonstrates the importance of chromatin modifications in signalling of DNA damage and making the repair pathway choice.

### Histone acetylation and DSB repair

The various post-translational modification of histones at the DSB can act as a barrier *via* compaction or can make chromatin accessible via decompaction during the process of damage signalling as well as repair (Aleksandrov et al., 2020). Acetylation of histones is one such dynamic chromatin modification regulated by the concerted action of HAT and HDAC (Gong and Miller, 2013; Roos and Krumm, 2016). Acetylation of lysine residues changes the charge at the DNA-nucleosome interface, leading to more open and accessible chromatin (Dhar et al., 2017). The histone acetyltransferases can be grouped into five subfamilies, namely HAT1/KAT1 (yHAT1), Gcn5/PCAF (yGcn5, hGCN5, hPCAF), Myst (yEsa1, ySas2, hMOZ, hMOF, hTIP60, etc), p300/CBP (hp300, hCBP), and Rtt109 (yrtt109) (Carrozza et al., 2003; Utley and Cote, 2003). Among these, p300/CBP subfamily is metazoan specific while Rtt109 is yeast specific (Marmorstein and Zhou, 2014). Histone acetylation is reversed by histone deacetylases, an action that restores the positive charge of the lysine. There are four classes of HDAC: Classes I and II contain enzymes that are most closely related to yeast scRpd3 and scHda1, respectively, class IV has only a single member, HDAC11, while class III (referred to as sirtuins) are homologous to yeast scSir2 (Glozak and Seto, 2007). This latter class, in contrast to the other three classes, requires a specific cofactor for its activity, NAD+ (Glozak and Seto, 2007; Greiss and Gartner, 2009).

The acetylation modifications at the N-terminus of the histones are most commonly studied as they are highly accessible at the tails and mediate binding of reader chromatin proteins (Kouzarides, 2007; Soria et al., 2012). Five reversible acetylable lysines are present at the N-terminus of histone H3 namely, 9, 14, 18, 23, and 27, whereas four acetylable lysines are present at positions 5, 8, 12, and 16 at the N-terminus of Histone H4 (North and Verdin, 2004; Wang et al., 2007; Ma and Schultz, 2008; Hulin et al., 2016; Khilji et al., 2021; Song et al., 2021; Wu et al., 2022). Interestingly, covalent modifications also occur within the globular domain of histones, especially at positions that are in close contact with the nucleosomal DNA wrapped around each octamer. One example of such acetylation is histone H3 lysine 56 (H3K56ac). The other histones like the linker H1 and the H2A are also modified at lysines with important roles in DSB repair. Table 1 shows the list of all histone lysine acetylation modifications which are regulated in response to DSBs**.** There are mainly three ways by which lysine acetylation of histones act at the chromatin- Activation of DDR pathway via making chromatin accessible, helping the remodelling of chromatin around DSB to help in DNA repair factor mobility and localization and lastly restoration of chromatin post DNA repair through inactivation of checkpoint (for example, H3K56ac is required for inactivation of checkpoint, also described in H3K56ac section) and later nucleosome packaging to native chromatin state (Figure 1). The bromo-domain (BRD) containing proteins act as the reader of acetylation marks through which many repair proteins come to the site of damage and thereby mediate repair functions (Marmorstein and Zhou, 2014). The role of histone acetylation and deacetylation in DSB repair was indicated by some of the earliest studies where the HDAC, sirtuins were shown to play a role in recombinational repair. The mutants of Sir proteins and Rad52 were shown to be synthetically sensitive to gamma-irradiation (Tsukamoto et al., 1997). Using two Hybrid assay, the Sir2, Sir3, and Sir4 were found to physically interact with Ku, the NHEJ factor (Tsukamoto et al., 1997). Consistent with this, Sir2 along with other sir proteins relocalize to the site of damage and help in silencing as well as chromatin compaction (Martin et al., 1999; Mills et al., 1999; Guarente, 2000). These proteins relocalize to the sites of damage along with the NHEJ protein Ku (Martin et al., 1999). Subsequently, indications on the role of histone acetylation in DSB repair came in the early 2000. The acetylation of histone H4 at the N-terminus residues catalysed by Esa1 acetyltransferase (mammalian Tip60 homolog) was first shown to be implicated in DSB repair (Bird et al., 2002). Early studies (Qin and Parthun, 2002) and (Tamburini and Tyler, 2005) have shown the role of histone H3 acetylation catalysed by Hat1 acetyltransferase and Gcn5 in the repair of DSB induced by the mating-type switching HO endonuclease. In support of these studies, the deletion of acetyl transferases responsible for the acetylation of these histone residues such as Tip60 in mammals, NuA4 subunit yng2 and gcn5 were also found to have DSB repair defects, genome stability functions, tumor suppressor functions, consistent with the roles of acetylation in DSB repair (Ikura et al., 2000; Choy and Kron, 2002; Kusch et al., 2004; Gorrini et al., 2007; Sun et al., 2010). The first direct evidence on the role of histone acetyltransferases in DSB repair came from the localization studies of Nua4 and Tip60 at the chromatin near DSB (Downs et al., 2004; Murr et al., 2006). NuA4/Tip60 is recruited to DSBs to acetylate Histone H4, H2A as well as H2AX and facilitate chromatin opening (Sun et al., 2010; Jacquet et al., 2016). It also has non-histone targets such as ATM which facilitates the DSB repair signalling (Sun et al., 2005). The human HATs like Mof1, TIP60, CBP, p300, and GCN5 play redundant roles in regulating acetylation at DSBs. Interestingly, ablation of CBP, p300, and Tip60 lead to decreased NHEJ (Van and Santos, 2018). Analysis using experiments such as laser microirradiation and ChIP at I-Sce1 induced DSBs, these acetyltransferases were found to be accumulated at the sites of DSB along with γ-H2AX and NHEJ factors Ku70, Ku80, 53BP1 (Murr et al., 2006; Ogiwara et al., 2011; Jacquet et al., 2016). The histone acetylation and deacetylation landscapes dictate the choice of pathway for repair of DSBs. For example, the histone acetylation mark H4K16 has been shown to counteract binding of 53BP1 leading to resection and repair by HR (Tang et al., 2013). Tip60 mediated H2AK15ac also leads to inhibition of 53BP1 binding at DSBs (Jacquet et al., 2016). These epigenetic landscapes are therefore dynamic and becomes crucial when the DSBs occur during the process of other DNA metabolic activities such as DNA replication, transcription, etc. (Aleksandrov et al., 2020). This review will here on focus majorly on histone H3 and H4 acetylation in DSB signalling and repair with crosstalks with other modifications.

**TABLE 1:** List of acetyl lysine modifications of Histones with roles in DSB signaling and Repair.

Histone acetylation	Acetyl transferase	Function in DDR	Reference
H1K85ac	PCAF	Decreases immediately post DNA damage. Promotes heterochromatin protein 1 (HP1) recruitment leading to condensed chromatin	Li et al. (2018)
H2AK15ac	Tip60	Peaks at S/G2, reduces at sites specifically repaired by NHEJ. Tip60 dependent H2AK15ac regulates DSB repair pathway choice by inhibiting H2AK15Ub and binding of 53BP1 thus, promoting HR.	Jacquet et al. (2016)
H2AX K5ac	TIP60	Decreases the spread of γH2AX-P upon damage. Aids in NBS1 accumulation at the damaged regions via H2AX exchange, thus aiding in ATM signalling	Kusch et al. (2004); Ikura et al. (2007); Jha et al. (2008); Ikura et al. (2015)
H2AX K36ac	p300/CBP	Constitutive acetylation, does not increase on radiation damage, however, promotes IR survival independently of gH2AX phosphorylation	Jiang et al. (2010)
H2BK120ac	SAGA acetyl transferase	Upon DSB induction H2BK120ub to H2BK120ac switch occurs irrespective of the region of DSB. May help in nucleosome remodelling	Clouaire et al. (2018)
H3K9ac	GCN5, PCAF	Reduces upon DNA damage, helps in localization of Swi/SNF complex to γH2AX containing nucleosomes. Obstructs ATM activation in stem cells leading to IR sensitivity	(Tjeertes et al., 2009; Lee et al., 2010; Meyer et al., 2016)
H3K14ac	GCN5	Increases in response to damage, helps in localization of Swi/SNF complex to γH2AX containing nucleosomes. Stimulated by HMGN1 and required for the activation of ATM.	(Kim et al., 2009; Lee et al., 2010)
H3K18ac	p300/CBP, GCN5	Recruitment of SWI/SNF and Ku at initial timepoints during G1 phase, later deacetylation by Sirt7 leads to loading of 53BP1 to facilitate effective NHEJ.	(Ogiwara et al., 2011; Vazquez et al., 2016; Swift et al., 2021)
H3K56ac	p300/CBP	Both reduction and increase observed post DNA damage, Deacetylated by Sirt6 and Sirt3 promotes NHEJ by recruiting SNF2H and 53BP1 to the DSB sites. Deactivates checkpoint to facilitate recovery and chromatin assembly	(Chen et al., 2008; Das et al., 2009; Tjeertes et al., 2009; Miller et al., 2010; Vempati et al., 2010; Battu et al., 2011; Toiber et al., 2013; Clouaire et al., 2018; Sengupta and Haldar, 2018; Vadla et al., 2020)
H4K5ac, H4K8ac	Tip60-Trap	Repair by HR by facilitating recruitment of MDC1, BRCA1. 53BP1, RAD51	(Murr et al., 2006; Ogiwara et al., 2011; Clouaire et al., 2018)
H4K12ac	p300/CBP	Recruitment of SWI/SNF complex, KU70/80 and repair by NHEJ	
		H4K12ac was reduced at AsiSI induced DSBs	
H4K16ac	Tip60-Trap MOF1	Biphasic response at the DSBs, facilitates both NHEJ and HR. Initial decrease and then increase at later timepoints. Abrogation of MDC1, 53BP1 and BRCA1 foci in the absence of MOF1	(Li et al., 2010; Miller et al., 2010; Sharma et al., 2010)

### Histone H4 acetylation and DSB repair

The role of histone H4 acetylation in the regulation of transcription by opening up chromatin is well known. However, the deletion of enzyme responsible for H4 acetylation, the human Tip60 lead to defective DSB repair capacity post IR treatment suggested the functions of histone H4 acetylation in DSB repair pathway (Ikura et al., 2000). The TIP60 acetyltransferase subunit, acetylates histone H4 at K5, K8, K12, and K16, as well as H2A at K5 and K15 at the DSBs. Histone H4 acetylation reduces the charge dependent histone-DNA interactions and also provides a platform for landing of a class of chromatin proteins that contain bromodomains (Umehara et al., 2010; Plotnikov et al., 2014; Gong et al., 2016). Of the potential H4 acetylation sites, the levels of H4K16ac increase after DNA damage and absence of H4K16ac leads to defective DNA repair (Li et al., 2010; Miller et al., 2010; Sharma et al., 2010). The Myst family acetyltransferase MOF1 catalyses H4K16ac. Upon deletion of MOF1, defective recruitment of MDC1, 53BP1 and BRCA1 was observed at DSBs (Li et al., 2010; Sharma et al., 2010). Reduced MDC1 in MOF1 deletion leads to reduced activation of ATM (Gupta et al., 2005). MOF1 mediated H4K16ac facilitates interaction with acidic patch of H2AX for recruitment of MDC1 and other chromatin remodelling events facilitating effective DNA repair (Figure 2) (Dhar et al., 2017). The histone chaperone Asf1 interacts with human MOF1 and regulates ATM activation via H4K16ac (Huang et al., 2018b). Asf1 also helps in NHEJ by mediating the phosphorylation of MDC1 by ATM (Lee et al., 2017). Given the role of H4K16ac in activation of ATM i.e., the sensing and signalling step of DSB repair, the H4K16ac kinetics at DSBs and its role in repair is however complicated. Whether acetylation has a positive role in DNA damage repair is still unclear. In budding yeast, Sin3 and Rpd3 dependent deacetylation of H4K16 at the DSBs regulate repair by NHEJ (Jazayeri et al., 2004). Similarly, in mammalian cells, after laser-induced DNA damage, H4K16Ac levels decrease rapidly followed by a steady increase at DSBs (Miller et al., 2010). The deacetylation of H4K16 was coincident with localization of HDAC1 and HDAC2 at the damage sites at initial time points. Depletion of both HDAC1 and HDAC2 results in hyper-acetylation of H4K16Ac and defects in NHEJ in humans as well as mice (Miller et al., 2010). H416ac presents as an obstacle in formation of higher order chromatin structure even though it increases chromatin accessibility. The deacetylation of H4K16ac leads to chromatin compaction which might be required to create a microenvironment for quick access and recruitment of NHEJ factors to the DSB site (Fernandez-Capetillo and Nussenzweig, 2004). The biphasic response of H4K16ac in response to DSB could be due to its role in regulation of DNA repair pathway choice. DNA repair by NHEJ can occurs fast anytime while HR is the preferred pathway only when the sister chromatids are available for repair i.e., specifically in S/G2 phase of the cell cycle and it is slower as compared to NHEJ. The major factor responsible for initiating NHEJ is recruitment of 53BP1 which inhibits DNA end resection. Studies using Nuclear Magnetic Resonance (NMR) and peptides containing specific histone marks has found, acetylation of H4K16 to be inhibitory toward the binding of the tudor domains of 53BP1 to H4K20me2 (Figure 3). Also, the HAT Tip60 has been implicated in the accumulation of BRCA1 at the chromatin while inhibiting 53BP1. The Tip60 complex also binds to H4K20me2 (through the MBTD1 complex) and prevents ubiquitination of H2A by directly acetylating the H2AK15 ubiquitin site, providing an example of how acetylation of a specific residue can inhibit other modification at the same residue (Figure 3). This Tip60-H4K20me2-H2AK15Ub-Ac axis helps promote HR by inhibiting 53BP1 (Tang et al., 2013; Jacquet et al., 2016). The role of H4 acetylation in regulating BRCA1 recruitment is also supported by another recent study where in S/G2 phase, the acetyl CoA generating enzyme ACLY is phosphorylated in response to DSB and leads to H4 acetylation by Tip60 which further recruits BRCA1 (Sivanand et al., 2017). BRDs act as lysine readers at the chromatin and have significant roles in DSB repair (Figure 3). Several BRD proteins like BRD4, ZMYND8, ACF1, TRIM28 (KAP-1), and TRIM33 are recruited to DSBs (Chiu et al., 2017; Gong and Miller, 2018). Some HATs such as p300 and GCN5 also possess BRD domains. CBP/p300 localizes to DSB sites and acetylates H4 at K5, K8, K12, and K16 and this leads to recruitment of NHEJ protein Ku70 and Ku80 to the sites of DSB (Ogiwara et al., 2011). These acetylations also help establish chromatin remodeling events at the break sites by enabling recruitment of SWI/SNF complex (Ogiwara et al., 2011). Recently, it was shown that H4K12ac was significantly reduced at AsiSI induced DSBs (Clouaire et al., 2018). Therefore, the molecular functions of this modification still remains to be explored further. In summary, H4ac in crosstalk with other histone modifications and readers can act as a barrier for the NHEJ pathway, while promotes HR and this dictates the pathway choice for DSB repair. For more detailed overview of Histone H4 acetylation and DSB repair, we refer the readers to other reviews which are specifically on role of histone H4 acetylation (Gong and Miller, 2013; Dhar et al., 2017).

### Histone H3 acetylation and DSB repair

The exact role of histone H3 acetylation in DSB repair pathway is less understood. Consistent with the role of acetylation in making chromatin accessible, it was suggested that histone H3 acetylation is required for the recruitment of the SWI/SNF complex in cooperation with γ-H2AX to DSB sites which promotes further nucleosome remodeling to mediate repair (Downs et al., 2004; van Attikum et al., 2004; Lee et al., 2010; Ogiwara et al., 2011).

DNA damage-induced changes in acetylation of mammalian histone H3 N-terminal lysines 9, 14, 18, 23 and 56 was observed by several studies (Das et al., 2009; Tjeertes et al., 2009; Yuan et al., 2009; Lee et al., 2010; Miller et al., 2010; Vempati et al., 2010; Guo et al., 2011). The dynamic nature of acetylation and deacetylation at H3K9, 14, 18, 23, 27 in response to a DSB created by a HO endonuclease was established first by earlier studies (Tamburini and Tyler, 2005), (Lee et al., 2010), where it was shown that histone acetylations at certain residues were first reduced and then increased to support repair and restoration. Additionally, it was shown that the acetylation was not only altered at the site of DNA lesion but also was altered at the donor locus or the sister chromatid. Consistent with this, the acetyl transferase responsible for H3 acetylation like Gcn5 and Esa1 and the histone deacetylases responsible for removal of acetyl mark for example, Rpd3, Sir2, and Hst1 were shown to be localized to the double-strand break during DNA repair (Jazayeri et al., 2004; Tamburini and Tyler, 2005). Histone acetylation marks such as histone H3 at lysine 56 (H3K56ac) is known to be associated with open chromatin. However, on the contrary a prevailing view suggests deacetylation of H3K56 is an early event in the response to DSBs. Certain histone acetylation marks such as H3K56 and H4K16 get activated in phases or waves, showing initial reduction and later on increase at the sites of DSB which indicates the dynamic role of both HATs and HDACs in sensing as well as repair of DSBs.

### Histone H3K14ac and DSB Repair

H3K14ac, the H3 tail modification is known to be associated with transcriptionally active chromatin. H3K14ac along with other H3 and H4 tail modifications was first shown to be altered upon DSB repair at an HO endonuclease site triggered by homologous recombination pathway (Tamburini and Tyler, 2005). However, the specific role of H3K14ac in DSB repair is not defined. In fission yeast, H3K14ac is regulated by GCN5 and MST2 acetyl transferases (Wang et al., 2012). The combined deletion of gcn5, mst2 or the mutation of H3K14R (hypo-acetylation mimic) leads to severe sensitivity phenotypes in response to variety of DNA damage-inducing agents such as UV light, bleomycin, MMS (methylmethane sulfonate), and ionizing radiation. H3K14ac is induced at an HO endonuclease DSB site, indicating its active role at the DSB signalling or repair. Consequently, loss of H2A phosphorylation was observed in H3K14R mutant due to the compact chromatin structure and the accessibility of RSC complex was found to be reduced in fission yeast (Wang et al., 2012). In support of this, the RSC complex through its bromodomain regions was shown to be recruited to the chromatin via H3K14ac in budding yeast (Kasten et al., 2004). Further studies show the role of yeast RSC complex in facilitating the recruitment of ATM/ATR complexes (Tel1/Mec1) to the break site and for the induction of phosphorylation of H2A (Liang et al., 2007; Shim et al., 2007). Consistent with the roles of H3K14ac in DDR in yeast, H3K14 was found to be increased in response to IR treatment in mammalian cells in GCN5 dependent manner (Lee et al., 2010). H3K14ac is correlated with active chromatin (Wang et al., 2008). Since, H3K14 is a tail modification, it is downregulated by deletion of a nucleosome binding protein HMGN1. This axis of HMGN1-H3K14ac induces the activation of ATM *via* ATM autophosphorylation in response to IR (Figure 2) (Kim et al., 2009). The role of HMGN1 in the activation of ATM is due to the global reorganisation of ATM in the nucleus via H3K14ac and not due to local changes in interaction of ATM with HMGN1 or with other chromatin factors (Kim et al., 2009). This is a classic example of the role of histone H3 tail modification in the global nuclear changes leading to DDR signal activation. The specific role of H3K14ac in the DDR pathway is still emerging. Acetylated histones are read by bromodomain containing proteins. Recently, a bromodomain containing protein ZMYND8 is shown to localize to the sites of DSB (Gong and Miller, 2018). Independently, it was shown that ZMYND8 interacts with H3K14ac mark along with H3K4-me1 to regulate transcription of malignant genes (Li et al., 2016). Whether this axis of ZMYND1-H3K18ac is linked to the DDR signalling or repair can be checked in the future. Also, the detailed kinetics of H3K14ac using laser induced site specific damage is needed to further gain knowledge about the specific signalling events orchestrated by this H3 tail modification leading to repair. Since, its crosstalk with other histone modifications in regulating transcription is known, whether this is true for DSB repair could be an interesting question to pursue in the future.

### Histone H3K18ac and DSB repair

Several studies reported H3K18ac, one of the histone mark of the N-terminus of histone H3, at the site of DSBs (Schiltz et al., 1999; Tamburini and Tyler, 2005; Ogiwara et al., 2011; Vazquez et al., 2016). p300 and CBP dependent H3K18ac mediates the access of the chromatin remodeling complex SWI/SNF to the DSB site (Ogiwara et al., 2011). Furthermore, DNA damage caused by ionizing radiation resulted in GCN5-mediated H3K18ac. Further, this modification along with acetylation marks at other N-terminal residues in H3 is induced on γH2AX containing nucleosomes leading to the binding of BRG1, the ATPase subunit of SWI/SNF complex (Figure 2). This mechanism helps in spreading of phosphorylation of H2AX on nucleosomes flanking the DSB and thus forms a feedback loop to facilitate DSB repair (Lee et al., 2010). However, the kinetics of H3K18ac at the DSBs was unclear. Recently, interesting details emerged about the kinetics of H3K18ac levels at the DSBs. A rapid increase in H3K18ac was observed post 15 min of IR treatment followed by reduction and this reduction persisted till the end of repair (Vazquez et al., 2016). Incidentally, in response to IR and genotoxic stress, the sirtuin SIRT7 is recruited to DSB sites as early as 1 s and peaks at 1 min to mediate deacetylation of H3K18ac and this fine-tuning is required for the binding of 53BP1 to the chromatin and making an early choice for NHEJ (Figure 3). This loading of SIRT7 to the chromatin is ATM-independent and is dependent upon the sensor PARP (Vazquez et al., 2016). Consistently, the NHEJ efficiency was significantly reduced in SIRT7 knock out cells. SIRT7 deficiency also leads to replication defects and fork collapse. This suggests that H3K18ac role at the chromatin may not be limited to DSB repair in G1. Coincidently, a very recent report introduced a new player, a transcription factor SP1 in the regulation of H3K18ac *via* p300 (Swift et al., 2021). SP1 is required to recruit p300 to the DSB site during G1 phase and induce H3K18ac. The induced H3K18ac is required for recruitment of SWI/SNF complex and NHEJ factor Ku to the DSB. These results at first seem contradictory to the earlier study where deacetylation of H3K18ac is required for NHEJ factor 53BP1 binding. It is therefore hypothesized that, initial opening of chromatin *via* H3K18ac mediated by p300 and SP1 is required for initiating the NHEJ pathway in G1 phase by recruitment of SWI/SNF and Ku80. Further, deacetylation by SIRT7 could be required for 53BP1 loading to restrict resection and finishing DNA repair (Figure 3). In support of this, it was reported that Dicer is upregulated in response to DSBs which sequesters SIRT7 in the cytoplasm at the early timepoints to facilitate chromatin opening via H3K18ac (Zhang et al., 2016; Chen et al., 2017). Subsequent release of SIRT7 leads to deacetylated H3K18 promoting effective repair by NHEJ. Clearly, the role of H3K18ac in the DSB repair pathway needs further investigation. The global role of H3K18ac in the regulation of transcription is known. However, fine tuning the levels of this acetylation at a particular DSB in different cell cycle stages is crucial to mediate the repair.

### Histone H3K56ac and DSB repair

Acetylation of the globular domain residue, histone H3K56 in the alpha N helix that is strategically positioned at the DNA entry and exit site in the nucleosome, was first discovered in budding yeast by mass spectrometry (Masumoto et al., 2005; Xu et al., 2005). Structurally, H3K56 faces the major groove of the nucleosomal DNA providing an excellent position to affect histone/DNA interactions when acetylated (Davey and Richmond, 2002; Gershon and Kupiec, 2021). The histone H3K56 is acetylated in the S-phase of the cell cycle specifically behind the replication forks and is deacetylated by the sirtuins at G2/M phase. In yeast, all newly synthesized histone H3 in S phase are acetylated at H3K56 residue (Hyland et al., 2005; Masumoto et al., 2005; Ozdemir et al., 2005; Xu et al., 2005; Xhemalce et al., 2007; Haldar and Kamakaka, 2008). The histone H3K56ac is conserved in mammals and is associated with human cancers (Das et al., 2009). Acetylation of H3K56 leads to increased DNA accessibility by facilitating spontaneous unwrapping at the entry and exit points of the nucleosome. This is supported by many biophysical studies (Neumann et al., 2009; Kim et al., 2015). H3K56ac is regulated by CBP/p300 in humans along with histone chaperone Asf1a and is deacetylated by HDAC1/2, sirtuins, SIRT1, SIRT2, SIRT3, and SIRT6 (Das et al., 2009; Yuan et al., 2009; Vempati et al., 2010).

### Role of H3K56ac in yeast DSB repair

The yeast acetyltransferase Rtt109 acetylates H3K56 in collaboration with the chaperones, Asf1 (a H3-H4 chaperone) and Vps75 (Celic et al., 2006; Schneider et al., 2006; Driscoll et al., 2007; Han et al., 2007; Tsubota et al., 2007). Asf1, in complex with H3K14ac-H4, alters the selectivity of Rtt109-Vps75 significantly towards H3K56ac, indicating crosstalk among different H3 acetylations (Cote et al., 2019). The sirtuins ScHst4 and ScHst3 in *S. cerevisiae* and the SpHst4 in *S. pombe* regulate cell cycle progression and heterochromatin silencing and assembly (Brachmann et al., 1995; Freeman-Cook et al., 1999; Haldar and Kamakaka, 2008; Konada et al., 2018). These deacetylases remove and thus, negatively regulate H3K56ac levels during the cell cycle as well as post DNA damage. Several studies showed this modification is required for maintenance of genome integrity (Celic et al., 2006; Maas et al., 2006; Miller et al., 2006; Xhemalce et al., 2007; Haldar and Kamakaka, 2008). The acetylated histone H3K56 promotes replication-coupled nucleosome assembly as well as assembly of nucleosomes following repair by increasing interaction between histone chaperon CAF1, Rtt106 and Asf1 and histones (Chen et al., 2008; Li et al., 2008). This is required for restoration of chromatin structure following DNA replication or repair, as has been depicted by several studies and proposed in the access-repair-restore model, a necessary step for maintenance of genome integrity (Green and Almouzni, 2002). Interestingly, the levels of H3K56ac are maintained in response to DNA damage during S-phase in the checkpoint dependent manner (Masumoto et al., 2005; Thaminy et al., 2007). The tight regulation of this modification is via the downregulation of sirtuins Hst3 and Hst4 in S-phase and post-DNA damage in S-phase (Celic et al., 2006; Haldar and Kamakaka, 2008). Budding yeast Hst3 is regulated by CDK dependent phosphorylation and degradation via SCF (Cdc4) ubiquitination pathway (Delgoshaie et al., 2014; Edenberg et al., 2014). Checkpoint sensor kinase Mec1 regulates Hst3 levels in an intra-S-phase checkpoint kinase Rad53 dependent mechanism (Thaminy et al., 2007). In fission yeast, *S. pombe,* SpHst4 which is the functional homolog of budding yeast hst3, hst4, has also been recently shown by our lab to be degraded in an ubiquitin dependent manner (Aricthota and Haldar, 2021). Notably, the DDK kinase Hsk1 phosphorylates Hst4 at the C-terminus in response to DNA damage caused by methylmethane sulfonate treatment, which thereby is recognized by the SCF (Pof3) complex and ubiquitinated. Hst4 is then targeted for degradation via proteasome. In response to DNA damage caused by methylmethane sulfonate (MMS) treatment.

Deletion of sirtuins Hst3 and Hst4 induces several genome instability phenotypes, including spontaneous DNA double-strand breaks, increased chromosomal loss, impairment of break-induced replication, and increased sensitivity to genotoxic agents (Brachmann et al., 1995; Freeman-Cook et al., 1999; Che et al., 2015). Notably, these phenotypes are suppressed by deletion of histone chaperone Asf1 which is essential for the activity of Rtt109 histone acetyltransferase (HAT) complex or by a non-acetylatable H3K56R mutant, suggesting that constitutive H3K56 hyperacetylation results in genomic instability (Celic et al., 2006; Maas et al., 2006; Driscoll et al., 2007). The absence of H3K56ac is equally harmful for the genome stability as expression of hypoacetylated H3K56R mutant or the absence of Asf1 leads to severe sensitivity in the presence of genotoxic agents such as, methylmethane sulfonate (MMS), campthotecin (CPT), and hydroxyurea (HU), etc., (Lewis et al., 2005; Maas et al., 2006; Haldar and Kamakaka, 2008). Inability to downregulate Hst4 of *S. pombe*, in a phosphorylation defective mutant of Hst4, 4SA-hst4 leads to hypoacetylated H3K56 and this mutant suffers sensitivity and defective recovery from replication stress (Aricthota and Haldar, 2021).

The genome stability defects observed upon perturbation of H3K56ac pathway indicates the role of H3K56ac in the regulation of DDR signalling or repair. Absence of sirtuins Hst3 and Hst4 leads to activation of checkpoint without any exogenous treatment indicating spontaneous and persistent DNA damage. Similar results were obtained in the absence of rtt109 deletion indicating that dynamic regulation of H3K56ac functions in the DDR pathway (Driscoll et al., 2007). Studies have indicated that rtt109, asf1 functions in the same pathway as asf1 in the response to genotoxic drug treatments (Recht et al., 2006; Driscoll et al., 2007). High levels of H2A phosphorylation was seen in cells with hyperacetylated as well as hypoacetylated genome (Simoneau et al., 2015; Aricthota and Haldar, 2021). Also, high recombination foci (Rad52 foci) were observed in the absence of exogenous damage in these cells, indicating cells with deregulated H3K56ac pathway face spontaneous DNA damage possibly due to defects in repairing the replicative DNA damage (Wurtele et al., 2012; Konada et al., 2018). The absence of H3K56ac in rtt109 and h3K56R conditions leads to persistent Rad51 foci which could be due to inability to resolve the damage downstream of Rad51. The DNA damage checkpoint gets deactivated once the damage is repaired i.e., during recovery. The absence of H3K56ac by deletion of Rtt109 or Asf1, leads to activated checkpoint post damage removal and due to this, cells are unable to re-enter cell cycle (Chen et al., 2008; Tsabar et al., 2016). This cell cycle re-entry mechanism is conserved in *S. pombe*, as the non-degradable phospho-mutant of hst4 (4SA-hst4) shows hypoacetylation of H3K56ac and defective recovery from replication stress (Aricthota and Haldar, 2021). These defects could be due to the role of chromatin reassembly functions of H3K56ac in deactivating the checkpoint.

The role of H3K56ac in HR in yeast is not established due to the absence of sensitivity of the mutants of this pathway in response to acute IR treatment. Also, it was observed that acute exposure to IR did not induce H3K56ac in *S. cerevisiae* (Masumoto et al., 2005). Further, cells lacking Rtt109 or Asf1 are capable of repairing a single HO-induced DSB. The genetic interaction data of *hst3hst4* mutants with the HR pathway genes in yeast suggests that Rad51 is not required for the survival of these mutants. However, the survival depends on the Rad52 and MRN complex (Munoz-Galvan et al., 2013). These data suggest that H3K56ac pathway is specifically required in a branch of HR repair which is not dependent on Rad51. One such example is the repair by break induced replication (BIR), which is needed to repair single ended DSBs (Che et al., 2015). Since, H3K56ac only occurs during S-phase of the cell cycle, it is assumed that it is not required for DNA repair activities outside S-phase. However, the H3K56R mutants were found to be sensitive to prolonged bleomycin treatment which induces DSBs and is repaired by Rad51 pathway, indicating that H3K56ac role in DSB repair still needs to be studied. In *S. cerevisaie*, H3K56ac has been implicated in the formation of meiotic breaks (Karanyi et al., 2019). Further supporting the possible role of H3K56ac in HR, the downregulation of Hst4 was also observed in response to MMS and HU but not in bleomycin treatment (which induce DSBs), indicating that only early S-phase fork stalling leads to induction of H3K56ac. The molecular role of Hst3/Hst4 and H3K56ac pathway in the DNA repair mechanisms induced by these damaging agents warrants further investigation. Also, it was shown in the fungus Neurospora, the role of H3K56ac and rtt109 in the regulation of Quelling and DNA damage-induced small RNA (qiRNA) production *via* homologous recombination. The H3K56ac was found at the site specific DSB break site in this study (Zhang et al., 2014).

H3K56ac is also required for the stability of advancing replication forks. Impairment of nucleosome assembly pathways through deletion of Asf1 or Caf1 leads to defective DSB repair during DNA replication (Lewis et al., 2005). Absence of asf1, rtt109 leads to increased recombination, as sister chromatid exchanges increase (Prado et al., 2004). The balance of acetylation and deacetylation of H3K56 during DNA replication is required to help the recombination machinery in choosing the right sister chromatid for the recombination during HR (Munoz-Galvan et al., 2013). Since, sister chromatid recombination is the major pathway for repair of replication induced DSBs, this could explain the sensitivity of H3K56ac pathway mutants to HU, CPT, etc., replicative stress causing agents. Overall, the accurate, timely and dynamic regulation of histone H3 lysine 56ac is the key to cell survival upon DNA damage.

### Role of H3K56ac in human DSB repair

The core domain modification, histone H3K56ac is conserved in mammals and is associated with human cancers (Das et al., 2009). It is regulated by CBP/p300 in humans along with histone chaperone Asf1a and is deacetylated by HDAC1/2, Sirtuins, SIRT1, SIRT2, SIRT3, and SIRT6 (Das et al., 2009; Yuan et al., 2009; Vempati et al., 2010). The role of H3K56ac in human DSB repair is a long studied question and still elusive. It is a DNA damage responsive modification as its level alter upon exposure to DNA damage. However, there are conflicting reports on the H3K56ac levels upon treatment of specific cell lines with same DNA damaging agents and therefore, the function and regulation of H3K56ac in DSB repair has been controversial in human. Some studies have shown that the level of H3K6ac increases in response to DNA damage (Das et al., 2009; Yuan et al., 2009; Vempati et al., 2010). However, other studies have shown that H3K56 is actively deacetylated at sites of DNA breaks (Tjeertes et al., 2009; Miller et al., 2010). Treatment of cells with PIKK inhibitors such as wortmanin leads to reduced H3K9ac and H3K56ac without any exogenous DNA damage (Tjeertes et al., 2009). This could be due to endogenous DNA damage induced by the inhibition of ATM/ATR kinases. The kinetics of reduction of H3K56ac is very fast and corresponds with the appearance of γH2AX upon treatment with Phleomycin. These seemingly contrasting results could be due to the non-specific signal by the different commercial antibodies available against H3K56ac or speculatively, could be due to difference cellular microenvironment i.e. the cell culture conditions which varied between these laboratories ad (Pal et al., 2016). The other reason for these contrasting results could be the growth conditions of the cell and its effect on the dynamicity of H3K56ac, where the initial level or the pre-exiting modification code/level would determine how the levels of this modification would alter. Recent results indicate that the cellular microenvironment plays a role in controlling the dynamics of HK56ac upon DNA damage in mammalian cells (Vadla et al., 2020). Specifically, the cell density changes and accumulation of metabolites and pH alterations affect the global levels of H3K56ac. Upon DNA damage, H3K56ac increases in low density cells with low initial acetylation, while acetylation decreases in high cell density cells. The gradual increase in H3K56ac from low to high cell density medium was coincident with decreasing levels of SIRT1 and SIRT6 (Vadla et al., 2020). Interestingly, unlike yeast, the global reduction of H3K56ac in response to DNA damage in humans is not dependent on cell cycle effects (Tjeertes et al., 2009). There are instances of similar changes in acetylation in response to damage due to the complex dynamics of DNA damage repair at the chromatin due to differences in DNA repair code generated due to subtle changes in cellular microenvironment. UV treatment leads to rapid hyperacetylation of all histones followed by a hypoacetylated state (Ramanathan and Smerdon, 1986). More recent studies have suggested this biphasic mode of H3K56ac where it decreases immediately upon DNA damage (UVR) and subsequently restored. Additionally, HDAC1 and HDAC2 act at DSBs to deacetylate H3K56ac to promote repair by NHEJ (Miller et al., 2010). The sirtuin, SIRT3 localizes to nucleus and deacetylates H3K56ac immediately to regulate NHEJ pathway via regulating recruitment of NHEJ protein 53BP1 (Sengupta and Haldar, 2018). This biphasic mode of post-translational modifications is interesting and has been observed for histone H4K16ac as well. Similar to H3K56ac, the linker histone H1K85ac is decreased immediately in response to IR treatment as well as at the site specific DSB to promote chromatin compaction, but increase at later timepoints (Li et al., 2018). H1K85ac promotes Heterochromatin protein 1 (HP1) recruitment at the chromatin which facilitates chromatin compaction. Reducing H1K85ac immediately post DNA damage by HDAC1 leads to chromatin decompaction. However, the role of H1K85ac in DSB repair is dynamic as both H1K85Q and H1K85R mutants are sensitive to IR treatments. HATs and HDACs function in regulating these dynamic modifications in order to remodel chromatin via recruitment of specific remodelers. Various chromatin remodelers, including INO80, the NURD complex, SMARCAD1, p400, CHD4, etc. were shown to be recruited to sites of damage, suggesting the need of chromatin remodeling in order to allow repair (Papamichos-Chronakis and Peterson, 2013; Price and D'Andrea, 2013; Xu and Price, 2011). Previous studies have shown that defects in DNA damage repair in SNF2H knockdown cells could be rescued with chloroquine treatment, a drug that causes chromatin relaxation (Murr et al., 2006; Nakamura et al., 2011). The NAD + dependent sirtuin, SIRT6 is required for the localization of SNF2H to the sites of DSB (Toiber et al., 2013). SIRT6 deacetylates H3K56ac at DSB to regulate SNF2H binding. It was observed that in the absence of SIRT6 and in H3K56Q mutants, SNF2H is unable to open chromatin leading to defective DSB repair signaling by inhibiting recruitment of repair proteins such as RPA, 53BP1, and BRCA1 (Toiber et al., 2013). Subsequent studies have linked SNF2H functioning downstream of RNF168- H2A ubiquitination pathway which regulates key steps in NHEJ at heterochromatic regions (Kato and Komatsu, 2015). Interestingly, SIRT6 also functions in regulating recruitment of another chromatin remodeler, CHD4 at the sites of DNA damage at G2 phase of the cell cycle, specifically at compacted DSB regions. SIRT6-CHD4 competitively binds H3K9me3 which helps in evicting the heterochromatin protein HP1 from the chromatin leading to chromatin decompaction to promote HR (Hou et al., 2020). Earlier reports have suggested that PARP dependent accumulation of CHD4 further recruits HDAC1/2 (Chou et al., 2010; Polo et al., 2010). Whether any other histone acetylation has function in this CHD4-HDAC1/2 pathway forming a repair code to regulate HR is not known. A study has however, shown that knock down of HAT p300 leads to reduced recruitment of CHD4 and their knock down independently lead to reduced HR while NHEJ was not affected. The fact that knock-down of both HATs like p300 and HDACs like sirtuins leads to defective DSB repair suggests the complex role of post-translational modifications in DSB repair. The histone chaperone Asf1 has been shown to regulate homologous recombination via enabling loading of Rad51 to the sites of DSBs (Figure 3) (Huang et al., 2018a). Also, similar to yeast, studies in mammalian cells have shown the role of H3K56ac in recovering from DNA damage via inactivating checkpoint, promoting chromatin reassembly and thus regulating cell-cycle progression (Chen et al., 2008; Battu et al., 2011). Since, Asf1, p300 and SIRT6 regulates H3K56ac, it is plausible to think that H3K56ac function in HR needs further detailed studies where cell cycle effects and time points are accounted for.

## Concluding remarks

DNA damage triggers a network of intricate signaling and repair mechanisms which take place in the chromatin context. Starting from detection of the lesion till the restoration of chromatin following repair, proteins involved in all steps of DNA damage response work in close coordination with the regulators of chromatin for making chromatin structure conducive for DDR and DNA repair. Histone modifications and modifiers alter chromatin by loosening contact with DNA thereby relaxing chromatin and recruiting DNA remodeling and repair factors via interaction with their bromodomain. Histones are acetylated on several residues and defect in acetylation of specific residue results in definite phenotypes. However, the molecular functions of these acetylation in DSB repair are not well understood. Further, crosstalk between several modifications are known and it has been proposed that these may form specific repair codes to determine downstream steps of repair pathways. Further research will through light on these mechanisms which will be crucial for understanding the complexities of DSB repair pathways and contribute to development of new therapeutics of diseases resulting from defective DSB repair.
